# Self-medication behaviors among Japanese consumers: sex, age, and SES differences and caregivers’ attitudes toward their children’s health management

**DOI:** 10.1186/1447-056X-11-7

**Published:** 2012-09-11

**Authors:** Ikuko Aoyama, Shinichi Koyama, Haruo Hibino

**Affiliations:** 1 Department of Design Science, Graduate School of Engineering, Chiba University, 1-33 Yayoi-cho, Inage-ku, Chiba-shi, Chiba, 263-8522, Japan

**Keywords:** Attitude toward OTC medicines, Health disparity, Medical disparity, Self-medication, Choice of OTC medicines

## Abstract

**Background:**

Since 2009, when the revised *Pharmaceutical Affairs Act* was enacted in Japan, self-medication practices have increased. Because the concept of self-medication was recently introduced in Japan, few studies exist on this topic. Therefore, it is necessary to explore how self-medication is practiced. This study examined Japanese consumers’ self-medication practices and attitudes toward over-the-counter (OTC) medicines based on their sex, age, and socioeconomic status (SES).

**Methods:**

The participants were 403 adults (*M*_age_ *=* 41.1 years, *SD* = 16.22). A quota sampling method was employed based on age group, and participants completed an online questionnaire.

**Results:**

Participants in the 20–29 age group reported medical costs as an obstacle in seeing a doctor; in contrast, transportation was a mitigating factor for elderly people. Regarding SES, people at lower SES levels chose to rest instead of seeing a doctor or purchasing over-the-counter (OTC) medicines when sick. They also placed more value on national brand OTC medicines than private brands (likely due to advertisements). This finding suggests individuals with a low SES do not select OTC medicines based on their effects or ingredients. Regarding attitudes toward OTC medicines, Japanese participants seemed to be unaware of the potential for abuse and side effects associated with OTC medicines. Finally, in relation to caregivers’ self-medication practices for their children, the majority of participants reported taking their children to the hospital since children tend to receive free medical care. Furthermore, caregivers with a high educational background are more confident in being able to help manage their children’s health.

**Conclusions:**

Our results suggest that health and medical discrepancies among Japanese consumers pose new social problems. In Japan, universal health care is available, but the cost of receiving medical care is not completely free of charge. Thus, we hope that the government will attempt to meet the various needs of patients and support their well-being. Consumers also have to be more independent and aware of their health management, as self-medication practices will continue to play a more significant role in healthcare. More research is needed to find ways to teach Japanese consumers/patients of both the benefits and risks of over-the-counter (OTC) medicines.

## Background

Self-medication, which is a common self-care practice, is widespread around the world [1]. Self-medication products account for approximately 20% of the total international pharmaceutical market [2]. Among various self-medication options, the use of over-the-counter (OTC) medicines is the most prevalent. In the U.K., an estimated £1,268.5 million was spent on OTC medicines in 1994, which equates to one-third of the total cost of prescribed drugs [3]. In Germany, self-medication sales were over £5.4 billion in 2006 [4]. The benefits of OTC medicines include convenience to consumers/patients, better self-management of minor problems, and a reduction in governmental medical costs. Thus, throughout the world, a wider range of medicines is becoming available directly to the public [2], and the current practice of self-medication offers consumers/patients more flexible choices in health management.

Self-care choices vary according to sex, age, and socioeconomic status (SES). For example, in Ireland, females report buying OTC medicines more often than do males, and younger adults report buying OTC medicines more often than older adults do [5]. With regard to SES, costs can function as a barrier that reduces access to prescription medicines [6], but the significance of this variable depends on the healthcare system within a particular country. In the U.S., where a universal health care system is unavailable, a study found that a significantly large number of mothers without health insurance were likely to give OTC medicines to their young children [7]. In contrast, in Denmark, a country with an established universal health care system, medical costs do not appear to function as a barrier that reduces access to prescription medicines [6]. In Japan, the effects of a long-term recession have negatively influenced the overall economic situation of many individuals. Thus, a medical/health discrepancy between people low and high in SES is apparent. Do people at lower SES levels hesitate to see a doctor because of medical costs? This question has yet to be well researched. When people at lower SES levels are encouraged to practice self-medication, are they able to choose medicines based on valid evidence of the medicine’s safety? Advertisements for OTC medicines are legal in many countries, including Japan, and such marketing might affect perceptions among consumers/patients as to the benefits of different products [2]. Traditionally, patients are rather passive and dependent, and they tend to follow the instructions of experts [1]. However, taking individual responsibility for safe self-medication practices is increasingly necessary as several OTC medicines become available on the market.

When discussing self-medication issues, the topic of self-medication for children is noteworthy. Research on this topic has been accumulating within Western countries for over a decade. In the U.S., 70% of illnesses among preschoolers were treated with non-prescription medicines [7]. However, the use of non-prescription medicines for children was often inappropriate. A U.S. study found that 71% of caregivers have inappropriately used non-prescription medicines for their children, and 10.9% reported that they only “sometimes” read labels and drug information [8]. Similarly, parents in the U.K. were generally unaware of potential side effects of OTC medicines because they believe that “over-the-counter medicines are not strong and were unlikely to harm their child,” or they believe that “prescription medicines are strong and otherwise carry risks” [9] p. 27]. On the other hand, Du and Knopf [4] conducted a profile analysis of caregivers who use OTC medicines and found “the higher the socioeconomic status of the children’s family, or the higher the educational level of the children’s mother, the more OTC medicines the children were likely to receive” (p. 606). Because OTC medicines for children are relatively new in Japan, few studies have been conducted. Therefore, it is necessary for public health policy makers to explore how self-medication is practiced for children in order to better construct parental education regarding OTC administration. Caregivers’ demographic information, along with their attitudes, must be addressed.

In Japan, the revised *Pharmaceutical Affairs Act* was only recently enacted in 2009; this introduced the concept of self-medication. In 2008, a group of Japanese researchers asked approximately 1,000 adults if they knew the term “self-medication” and 60.5% did not recognize this term [10]. However, research on self-medication issues is important given the fact that declining birthrates and a growing proportion of elderly people will have a significant impact on the Japanese pension and social welfare system in the near future.

Therefore, the goals of the present study were to examine the following: (1) how Japanese consumers/patients practice self-medication to manage their health problems, (2) how Japanese consumers/patients select particular OTC medicines, (3) how consumers/patients’ attitudes toward OTC medicines, and (4) caregiver-initiated medication behaviors for children. This study also investigated the role of sex, age, and socioeconomic status (SES) on the preceding research questions.

## Method

### Participants

Participants were 403 adults living in the Kanto area of Japan, which consists of seven prefectures including Tokyo (*M*_age_ = 41.12, *SD* = 16.19, range 20–79, male = 47.4%, female = 52.6%). The research company, Media Interactive Inc., recruited participants in 2011. Among the 403 participants, 331 had children.

A quota sampling method was employed to compare the following three age groups: a young group (20–29), middle age group (30–49) and a senior group (65 years old and above). There were an approximately equal number of male and female participants within each stratum (Table 1). These three age groups were selected for the following reasons. First, we wanted to examine how parents of young children (elementary school and younger) practiced self-medication for both their children and themselves. Thus, we placed a focus on assessing participants in their 30s and 40s. Second, we wanted to examine age differences in self-medication practices. These age groups (20s, 30 − 40s, and 65 and older) are especially important groups to address given the intergenerational economic inequality in Japan. According to a report from the Cabinet Office, Government of Japan (2012), the differences in net benefit (the ratio of net benefit from the social security system relative to lifetime income) between younger and older adults is 12% [11]. The government is acutely aware of the fact that the younger generation suffers a larger net burden and believes that intergenerational inequality should not be ignored. 

**Table 1:** Participants’ sex and age

	**n**	**%**
**Total (N)**	403	100.0
Male: 20s	61	15.1
Male: 30–40s	90	22.3
Male: 65–79	40	9.9
Female: 20s	82	20.3
Female: 30–40s	90	22.3
Female: 65–79	40	9.9

*Note:* There were no participants in the 50–64-year-old category.

### Procedures

All of the survey questions were uploaded online. The participants who agreed to complete the survey accessed it through a provided URL. After filling out the online survey, Media Interactive Inc. gave the participants points that could be accumulated in exchange for a gift card.

### Questionnaire and measures

Participants answered 43 questions, including information about demographics. The survey inquired about self-medication behaviors (for the individual and for children if the participant was a parent) and the reasons for their choice, the choice of OTC medicines, and attitudes toward OTC medicines (for the individual and for children if the participant was a parent).

### Self-medication behaviors

Self-medication behaviors were measured by the question: “When you have a high fever (39°C/102.2°F or higher), what do you do?” Response choices were (1) I will see a doctor and treat with prescribed drugs, (2) I will treat with OTC medicines, (3) I will rest for a while, (4) I will treat with an alternative method such as acupuncture and reflexology, or (5) Other. Next, participants were asked the reason for their choice (e.g., “Why do you see a doctor?” or “Why don’t you see a doctor?”). For their children, the question was, “When your child has a high fever (39°C/102.2°F or higher), what do you do?”

### The choice of OTC medicines

The choice of OTC medicines was measured by the question: “Which OTC medicines do you want to buy when you treat your illness?” Response choices were: (A) a national brand OTC medicine often seen in a TV advertisement or (B) a private brand OTC medicine that has the same effects and ingredients but is 50% cheaper than the national brand. National brand OTC medicines refer to medicines produced by a major pharmaceutical company (i.e., Tylenol and Advil in the U.S.). On the other hand, private brand OTC medicines refer to medicines that are similar to a national brand OTC medicine but are sold at specific retailers (i.e., Walmart). Examples of OTC medicines in Japan are aspirin and cough and cold medicines; antibiotics are not included.

In terms of reasons for selecting a national brand OTC medicine, response choices were (1) I feel safe because I often see the item on a TV advertisement, (2) I feel safe because a well-known pharmaceutical company produces it, (3) I feel safe because I have always taken the same medicine, (4) Cheap medicines make me feel anxious, (5) Unknown pharmaceutical companies make me feel anxious, (6) More expensive medicines seem to work better, (7) I like the package design, or (8) Other. Response choices for selecting a private brand OTC medicine were (1) It is cheaper, (2) Ingredients and effectiveness will be the same as the national brand OTC medicine, (3) There will not be a big difference among OTC medicines, (4) It seems better than the national brand sold by a major pharmaceutical company, (5) I like the package design, or (6) Other.

### Attitudes toward OTC medicines

Several questionnaires developed by Bradley et al., [3] Trajanovska, Manias, Cranswick, and Johnston [12], and Wazaify et al. [5] were adapted and translated for this study to measure patients’ attitude toward medicines. The questionnaire consisted of 23 questions measuring positive and negative attitudes toward OTC medicines, perceived value and side effects of OTC medicines, and criteria for purchasing OTC medicines. All questionnaire items and responses are listed in Table 2. 

**Table 2:** Patients’ attitude toward OTC medicines: questions and responses (%)

	**Strongly agree**	**Agree**	**Disagree**	**Strongly disagree**
The chemist is a good source of advice/information about minor medical problems.	14.9	67.2	15.6	2.2
A person should take medicines/treatment only when it is necessary.	60.3	36.5	2.2	1.0
You get good value from a doctor’s prescription.	23.1	66.0	10.7	0.2
Even if I have a health problem, I prefer to avoid taking any medicines.	17.4	35.7	39.5	7.4
We should be careful with non-prescribed OTC medicines.	18.4	52.6	27.8	1.2
People are less likely to bother their doctor with minor problems nowadays.	18.4	49.9	27.0	4.7
Only medicines/treatment from a doctor will really help.	3.7	18.4	57.3	20.6
Medicines/treatments you can buy are just as effective as those you get from a doctor.	3.0	18.4	57.3	20.6
Overall, seeing a doctor costs me less than buying OTC medicines.	7.7	43.2	39.5	9.7
Generally, I find the medicines you can buy are less effective.	7.4	38.7	48.6	5.2
More prescribed drugs should be deregulated to OTC status.	7.4	49.9	38.5	4.2
Prescription medicines are completely safe to use.	7.9	46.7	40.7	4.7
Non-prescription medicines can have dangerous side effects.	3.5	30.0	62.5	4.0
Non-prescription medicines can sometimes mask serious health problems.	8.2	50.9	39.0	2.0
Some non-prescription medicines interfere with the natural healing process of the body.	4.0	38.5	55.1	2.5
With continual use, some non-prescription medicines lose their effectiveness.	11.4	53.1	33.7	1.7
Some non-prescription medicines may cause dependency or addiction if taken for a long period of time.	8.2	47.1	40.9	3.7
Reading the label on the package is one of the ways I decide which medicines to take.	29.3	58.6	10.7	1.5
Advertisements help me to learn what types or brand of medicines you can buy.	3.7	39.7	47.6	8.9
I sometimes cannot afford to buy all the medicines I would like to buy myself (without a prescription).	10.9	32.5	44.7	11.9
I sometimes cannot afford to buy all the items on a prescription issued by a doctor.	4.0	16.9	58.6	20.6
If I am unsure about a problem, I always look for advice from a doctor or nurse.	25.6	48.1	23.6	2.7
I read the instructions carefully before taking a medicine or treatment for the first time.	27.3	46.2	23.8	2.7

### Caregiver-initiated medication behavior for children

Six questions from Maiman, Becker, Cummings, Drachman, and O’Connor [13] were used and translated to assess caregiver-initiated medication behavior for children. The questionnaire evaluated caregivers’ beliefs about their efficacy in managing the health of their children and their attitudes toward OTC medicines. All questionnaire items and responses are listed in Table 3. 

**Table 3:** Caregiver-initiated medication behavior for children: questions and responses (%)

	**Very true**	**Somewhat true**	**Somewhat not true**	**Not true at all**
Family is very often troubled by sickness.	7.1	23.4	56.0	13.5
I usually trust my own opinions about my child’s health more than a doctor’s opinion.	3.5	11.3	63.1	22.0
You have to use your own judgment in deciding how much of a doctor’s advice to follow.	11.3	61.7	24.1	2.8
I have been satisfied with prescription medicines I’ve given a child.	5.7	72.3	20.6	1.4
When I give a child an OTC medicine, I am as careful as I am when giving prescribed medicines.	22.0	56.7	18.4	2.8
I sometimes give a child OTCs in addition to those prescribed by a doctor for a problem.	2.1	22.0	34.0	41.8

### Socioeconomic status (SES)

SES was measured by annual income in Japanese yen. Possible responses were (1) less than 300 K, (2) 301–400 K, (3) 401–500 K, (4) 501–600 K, (5) 601–800 K, (6) 801–1000 K, (7) 1001–1500 K, or (8) 1501 K and above.

### Data analysis

Various chi-square (*χ*^2^) analyses were conducted to examine the presence of any differences in self-medication behaviors and the choice of OTC medicines according to sex, age, and SES. Phi coefficients (Φ) were calculated as measures of effect size. Phi values of 0.1, 0.3, and 0.5 may be interpreted as indicating a small, medium, and large effect between groups, respectively [14]. Standardized residuals within each cell greater or lesser than 1.96 were considered to be statistically significant at the .05 level. Subsequently, ANOVAs were conducted to examine which demographic variables were related to attitudes toward OTC medicines. There were no missing data, and SPSS 18.0 was used for all data analyses.

## Results

### Descriptive statistics

Participants’ demographic information is summarized in Tables 1 and 4.

**Table 4:** Participants’ income level

	**n**	**%**
**Total (N)**	403	100.0
Less than 300 K	72	17.9
301–400 K	73	18.1
401–500 K	67	16.6
501–600 K	50	12.4
601–800 K	65	16.1
801–1000 K	45	11.2
1001–1500 K	25	6.2
1501 K and more	6	1.5

Regarding descriptive statistics for self-medication practice, 65.8% of the participants chose, “(When I have high fever) I will see a doctor and treat with prescribed drugs.” When asked to explain their behavior, one-third of this group reported, “We are not health care professionals; thus, we should not make our own judgments.” The participants who chose, “I will treat the problem with OTC medicines” comprised 18% of the total group. When asked to explain their behavior, 20.3% of this group reported, “I want to treat the problem by myself,” and 19.6% reported, “First, I want to see if OTC medicines work for me.” For a summary of responses, see Tables 5, 6, 7.

**Table 5:** Summary of patients’ responses regarding self-medication behaviors

		**n**	**%**
**Total (N)**		403	100.0
1	I will see a doctor and treat with prescribed drugs.	265	65.8
2	I will treat with OTC medicines.	72	17.9
3	I will rest and see for a while.	63	15.6
4	I will treat with alternative methods such as acupuncture and reflexology.	3	0.7
5	Other	0	0.0

**Table 6:** Summary of patients’ responses regarding why they see a doctor

		**n**	**%**
	** Total (N)**	265	100.0
1	We use health care professionals; thus, we should not make our own judgments.	168	63.4
2	I trust a doctor.	43	16.2
3	Seeing a doctor is free of charge for me.	3	1.1
4	Prescribed drugs are cheaper than OTC medicines.	15	5.7
5	I feel comfortable seeing the same doctor.	31	11.7
6	Other	5	1.9

**Table 7:** Summary of patients’ responses regarding why they do not see a doctor

		**n**	**%**
	** Total (N)**	138	100.0
1	I have no time to see a doctor.	9	6.5
2	It costs money.	19	13.8
3	First, I want to see if OTC medicines work for me.	27	19.6
4	If I rest, it will be okay.	24	17.4
5	I want to treat the problem by myself.	28	20.3
6	I don’t like doctors.	1	0.7
7	I don’t like hospitals.	5	3.6
8	There are no hospitals nearby.	1	0.7
9	There is no transportation to get to a hospital.	2	1.4
10	It bothers me to go to see a doctor.	18	13.0
11	Other	4	2.9

However, when a child has a high fever, more than 90% of the caregivers would take their child to the doctor. The most common response was “We are not health care professionals; thus, we should not make our own judgments (63%).” The second most common response was “Medical costs are free for children” (12.6%).

With the regard to the question, “Which OTC medicines do you want to buy when you treat your illness,” 51.4% chose (A) a national brand OTC medicine, and 48.6% chose (B) a private brand OTC medicine. Logistic regression was performed in the selection of either a national brand or a private brand; however, none of the independent variables (age group, sex, and SES) showed significant group differences. Among respondents who chose a national brand, 13.5% reported “I feel safe because I often see the item on TV advertisements,” 27.0% reported “I feel safe because a well-known pharmaceutical company produces it,” 45.9% reported “I feel safe because I have always taken the same medicine,” and 13.5% reported “Unknown pharmaceutical companies make me feel anxious.” In contrast, when respondents who chose private brands were asked to explain their behaviors, about 50% reported “Ingredients and effectiveness will be the same as national brand OTC medicines,” and the other 50% reported “It is cheaper.” The comparison could not be made for children because only a few caregivers reported that they would use OTC medicines to treat their children’s high fever.

### Sex differences

A 2 (sex) × 5 (self-medication behavior choices) chi-square analysis did not reveal statistically significant differences, *χ*^2^ (3) = 2.18, *p* = 0.53, *n.s*. As for the choice of OTC medicines, no sex difference was found. The results suggest that participants’ self-medication behaviors and their choice of OTC medicines are not affected by their sex. This is partially consistent with findings reported by Hanibuchi [15], where no sex differences emerged in whether or not participants saw a doctor.

### Age differences

A 3 (age group) × 5 (self-medication behavior choices) chi-square analysis did not reveal statistically significant differences, *χ*^2^ (6) = 11.06, *p* = 0.08, *n.s.* A 3 (age group) × 2 (the choice of OTC medicines) chi-square analysis also did not reveal statistically significant differences, *χ*^2^ (2) = 0.88, *p* = 0.64, *n.s.* The results suggest that age group does not affect participants’ self-medication behaviors and choice of OTC medicines.

Subsequently, a 3 (age group) × 5 (reasons for not seeing a doctor) chi-square analysis revealed statistically significant age differences, *χ*^2^ (20) = 49.80, *p <* 0.01, Φ = .60. The phi coefficient indicated a moderate effect size. In examining the standardized residuals for each cell within the chi-square analysis, a significantly larger number of younger participants chose “(I am not going to see a doctor because) it costs money” as compared to the other two age groups. In addition, a significantly large number of senior participants chose “(I am not going to see a doctor because) there are no hospitals nearby” as compared to the other two age groups. In sum, younger adults are less likely to see a doctor because of financial constraints, and transportation issues can mitigate older adults’ ability/choice in seeing a doctor.

### SES differences

A 6 (SES group) × 5 (self-medication behavior choices) chi-square analysis did not reveal statistically significant differences overall, *χ*^2^ (21) = 21.89, *p* = 0.40, *n.s*.

However, in examining the standardized residuals for each cell within the chi-square analysis, results indicated that a significantly larger number of people in the “less than 300 K” group fell within the “will rest and see for a while” category as compared to the rest of the SES groups.

As for the choice of OTC medicines, a 6 (SES groups) × 2 (the choice of OTC medicines) chi-square analysis (*χ*^2^ (6) = 8.70, *p* = 0.19, *n.s*), a 6 (SES groups) × 5 (reason to pick private brand OTC medicines) chi-square analysis (*χ*^2^ (12) = 6.51, *p* = 0.88, *n.s*.), and a 6 (SES groups) × 5 (reason to pick national brand OTC medicines) chi-square analysis (*χ*^2^ (18) = 21.94, *p* = 0.23, *n.s*) did not reveal statistically significant differences. However, analyses of standardized residuals revealed that participants in the group whose annual income was less than 300 K were significantly more likely to fall into the “I feel safe because I often see the item on TV” and “Unknown pharmaceutical companies make me feel anxious” categories than the rest of the SES groups. In sum, people at lower SES levels are more likely to be influenced by advertisements when selecting OTC medicines. This is partially consistent with a U.S. study, which found that caregivers with a lower annual income (<US $20,000) are more likely to rely on packaging than caregivers who have a higher annual income (>US $ 60,000) [9].

### Attitude toward OTC medicines

An analysis of participants’ awareness of side effects provided interesting results. Thirty percent of the participants agreed, and 3.5% strongly agreed, with the statement “Non-prescription medicines can have dangerous side effects.” These are much lower percentages compared with the findings of a European study in which 59.7% of Irish respondents agreed and 5.9% strongly agreed with that same statement [5]. Similarly, only 38.5% of Japanese participants agreed with the statement “Some non-prescription medicines interfere with the natural healing process of the body,” as compared to 50.5% of participants in Ireland. Regarding the statement “Some non-prescription medicines may cause dependency or addiction if taken for a long period of time,” 47.1% of Japanese participants agreed compared to 65.2% of Irish participants. These results indicate that people in Ireland might be more aware of the potential abuse of some OTC medicines [5] as compared to Japanese participants.

### Caregiver-initiated medication behavior for children

The majority of caregivers (78%) were satisfied with the medicine they give to their children. Whereas 78.7% of the caregivers answered, “When I give child an OTC medicine, I am as careful as I am when giving prescribed medicines,” 23.1% reported “I sometimes give a child OTCs in addition to those prescribed by a doctor for a problem”; thus, the results are somewhat contradictory.

Caregiver-initiated medication behaviors for children were also analyzed based on caregivers’ age, SES, and educational level. Only education was a significant factor in a chi-square analysis: *χ*^2^(15) = 27.43, *p* < 0.05. In examining the standardized residuals for each cell within the chi-square analysis, caregivers who held a master’s degree or above were more likely to answer “very true” for “I usually trust my own opinions about my child’s health more than a doctor’s opinion.” In other words, caregivers with a higher educational background were more confident in managing their children’s health.

## Discussion

The goals of the present study were to examine the following: (1) how Japanese consumers/patients practice self-medication to manage their health problems and the reasons behind these practices, (2) how Japanese consumers/patients select particular OTC medicines, (3) how caregivers practice self-medication for their children, and (4) caregivers’ attitudes toward self-medication for their children. This study also investigated differences in self-medication management and attitudes across sex, age, and socioeconomic status (SES).

No significant sex differences were observed, but interesting age differences emerged; younger adults were less likely to see a doctor because of medical costs, and elderly adults were less likely to see a doctor because they lacked transportation. The younger-adult results partially support previous research that found that about 50% of younger adults (20s − 30s) were very anxious about their future medical expenses [16].

With regard to SES, participants within lower income levels were less likely to see a doctor or buy OTC medicines for their health problems. This result is also consistent with previous research showing that the number of individuals who do not see a doctor when they are sick because of costs more than doubles for individuals in lower SES groups as compared to those in higher SES groups [17]. However, Nielsen et al. [6] argue, “it is important that possible intervention by reimbursement systems is not socially imbalanced” [p. 199]. In Japan, universal health care is available, but the cost of medical attention is not completely free (as is the case in the U.K.). For people who receive governmental financial support in Japan, medical services dispensed at a hospital are often free. However, there is no equivalent financial support system helping those individuals acquire OTC medicines for free or at a reduced cost. Often, minor health problems can be treated with OTC medicines, but if these opportunities are inaccessible, medical attention might be postponed and problems can get worse. Consequently, patients might end up seeing a doctor and receiving long-term medical treatment, which would be more costly for the government relative to the cost of providing OTC medicines from the beginning. Advocacy for active self-medication has just begun during the past few years in Japan; thus, usage patterns for OTC medicines in terms of a cost-benefit analysis have yet to be assessed. Future research should pursue this topic. We hope that the government will attempt to meet the various needs of patients to support their well-being.

When assessing reasons why participants chose either national or private brand OTC medicines, results indicated that people in the lower SES groups placed more value on national brands than private brands. This was because they often saw national brands advertised on TV. These results suggest that OTC medicine advertisements have a powerful affect on perceptions among low SES consumers/patients. However, the influence of TV advertisements on public perceptions of OTC medicines has not been well researched. Advertisements on TV convey both intentional and unintentional messages to their audience, and consumers/patients should not rely solely on information garnered from such advertisements when choosing a product. Even though private brand OTC medicines are cheaper and have the same effects and ingredients as national brands, people among low SES groups prefer national brands; this might be because unknown pharmaceutical companies evoke some anxiety. In other words, low SES consumers might not choose OTC medicines based on relevant criteria, such as evidence of their effects or their ingredients. Even though major pharmaceutical companies produce some OTC medicines, consumers/patients still need to be aware of their potential adverse effects and use them as directed. Consumers/patients should consult a pharmacist to obtain more information, especially since pharmacists are easier to access than are doctors. A study in Ireland found that a recommendation by a pharmacist most frequently influenced consumer/patients’ choice of OTC medicines [5]. As self-medication practices become more common, it is important to be a wise consumer to improve one’s health and reduce medical costs. Therefore, pharmacists might play a more active role to help educate consumers/patients, especially since a large number of people do not pay attention to written information provided with medicines [18]. In fact, our results showed that 67.2% of participants agree with the statement “The chemist is a good source of advice/information about minor medical problems,” with 14.9% strongly agreeing. Some consumers/patients may not be ready to accept the more active role of pharmacists in their personal healthcare; however, pharmacists’ increasing involvement could lead to changes in public attitudes [3].

Our study also observed that Japanese participants were less aware of the potential for abuse and side effects associated with OTC medicines when compared to participants in a previous study conducted in Ireland [5]. Since the *Pharmaceutical Affairs Act* was recently revised and enacted in Japan, it is possible that Japanese consumers/patients are not highly aware of the risks associated with OTC medicines compared to Europeans. In fact, Kawase et al. [19] found that Japanese consumers/patients paid less attention to risk information labeled on OTC medicine packages than did Americans.

Finally, caregivers with higher educational backgrounds are more confident in managing the health of their children. As Du and Knopf [4] argue, “well-educated mothers often believe they have enough medical knowledge” [p. 607]. This trend is also consistent in Japan. However, we did not observe significant differences in parents’ efficacy in dealing with their children’s health based on SES. Given that medical costs for children are free in Japan, access to health care professionals is easy; thus, caregivers can take their children to the hospital if necessary. In fact, the majority of our participants reported they would take their sick children to the hospital instead of giving them OTC medicines. In addition, the second most common reason for taking their children to the hospital was “Free medical cost for children.” This suggests that the social welfare system for supporting children helps reduce caregivers’ anxiety.

## Conclusions

Our results suggest that individuals within different age groups and SES levels have different medical needs. This is noteworthy because dramatic changes in the Japanese social structure have occurred over the past few decades. The number of senior citizens is rapidly increasing while the birth rate remains low. In 2008, the birth rate was 1.37, a number that has not changed significantly over the past 10 years [20]. Meanwhile, people tend to live longer, and senior citizens now comprise 22.7% of the total Japanese population according to recent governmental statistics [21]. It is expected that senior citizens will comprise 33.7% of the total Japanese population by 2035, and this figure will increase to 40.5% by 2055 [22]. As a result, the financial burden placed upon younger adults will increase in order to support senior citizens in Japan. Consequently, younger adults might forfeit the opportunity to receive appropriate medical treatment from a doctor. The existing governmental healthcare system not match the current and future social structure. It is highly probable that more medical problems and need for treatment will emerge within the changing society; thus, we, as consumers/patients, have to change our mindset. The governmental social welfare system has functioned well, but it might not be as effective as it is currently in the future. To accommodate the changing social structure, people will need to become more independent in managing their health, and self-medication practice will likely play a key role.

Our results also suggest that more research is needed in order to find a way to teach both the benefits and risks of OTC medicines to Japanese consumers/patients. Along with public education, pharmaceutical companies can also improve the methods they use to deliver medical information. Raynor et al. [18] argue that risk information delivered numerically, rather than verbal descriptions, provides a more accurate estimation of the probability and likelihood of understanding a side effect or health risk. MacLennan & Sturdee [23] also suggest that all medicines should have a simple, visible labeling system on the outside of the package. Therefore, pharmaceutical companies might want to connect better with consumers/patients’ information processing systems by utilizing perspectives from cognitive psychology.

Finally, one limitation of this study is that it was cross-sectional in nature, which makes it difficult to infer causal relationships. Therefore, the generalizability of our findings is limited, and future research is required. Nevertheless, the present study brings a unique perspective to the literature by addressing differences in self-medication practices and attitudes according to sex, age, and SES among Japanese consumers/patients and caregivers.

## Competing interests

The authors declare that they have no competing interests.

## Authors’ contributions

IA carried out data collection, participated in the design of the study, and performed the statistical analysis. SK and HH participated in the study design and helped draft the manuscript. All authors read and approved the final manuscript.

## References

[B1] Chewning B Sleath B Medication decision-making and management: A client-centered model Soc Sci Med 1996 42 389 398 10.1016/0277-9536(95)00156-5 8658233

[B2] Bond C Hannaford P Issues Related to Monitoring the Safety of Over-The-Counter (OTC) Medicines Dr Safe 2003 26 1065 1074 10.2165/00002018-200326150-00001 14640771

[B3] Bradley PC Riaz ASR Tobias EJ Kentre Dassu YD Patient attitudes to over-the-counter drugs and possible professional responses to self-medication Fam Prac 1998 15 44 50 10.1093/fampra/15.1.44 9527297

[B4] Du Y Knopf H Self-medication among children and adolescents in Germany: results of the National Health Survey for Children and Adolescents (KiGGS) Brit J Clin Pharm 2009 68 599 608 10.1111/j.1365-2125.2009.03477.x PMC2780285 19843063

[B5] Wazaify M Shields E Hughes MC McElnay CJ Societal perspectives on over-the-counter (OTC) medicines Fam Prac 2005 22 170 176 10.1093/fampra/cmh723 15710640

[B6] Nielsen WM Hansen HE Rasmussen KN Patterns of psychotropic medicine use and related diseases across educational groups: national cross-sectional survey Eur J Clin Pharm 2004 60 199 204 10.1007/s00228-004-0741-4 15024533

[B7] Kogan DM Pappas G Yu MS Kotelchuck M Over-the counter medication use among US preschool-age children JAMA 1994 272 1025 1030 10.1001/jama.1994.03520130063034 8089884

[B8] Eiland L Salazar M English T Caregivers’ perspectives when evaluating nonprescription medication utilization in children Clin Ped 2008 47 578 587 10.1177/0009922807310244 18490663

[B9] Birchley N Conroy S Parental management of over-the-counter medicines Paed Nur 2002 14 24 10.7748/paed.14.9.24.s21 12510331

[B10] Narui K Suetsugu D Watanabe K Survey of consumer views on non-prescription drugs and self-medication before enactment of revised pharmaceutical affairs law in 2009 [In Japanese] JJPHCS 2010 36 240 251 10.5649/jjphcs.36.240

[B11] Suzuki W Masujima M Shiraishi K Morishige A Intergenerational inequality caused by social security systemhttp://www.esri.go.jp/jp/archive/e_dis/e_dis290/e_dis281.pdf

[B12] Trajanovska M Manias E Cranswick N Johnston L Parental management of childhood complaints: Over-the-counter medicine use and advice-seeking behaviours J Clin Nur 2010 19 2065 2075 10.1111/j.1365-2702.2009.03092.x 20920033

[B13] Maiman AL Becker HM Cummings KM Drachman HR O’Connor AP Effects of sociodemographic and attitudinal factors on mother-initiated medication behavior for children PHR 1982 94 140 149 7063595 PMC1424300

[B14] Green BS Salkind WJ Using SPSS for Windows and Macintosh: Analyzing and Understanding Data 2004 4 London: Pearson

[B15] Hanibuchi K Inequalities in health and health care access: Analysis of access to medical care using JGSS-2008 [In Japanese] JGSS Research Series 2010 10 99 110

[B16] Health and Global Policy Institute Public Opinion Survey on Healthcare Policy 2010 http://www.hgpi.org/handout/2010-02-08_06_973999.pdf

[B17] Health and Global Policy Institute Public Opinion Survey on Healthcare Policy 2007 http://www.hgpi.org/handout/2010-02-03_51_616703.pd

[B18] Raynor D Blenkinsopp A Knapp P Grime J Nicolson D Pollock K Spoor P A systematic review of quantitative and qualitative research on the role and effectiveness of written information available to patients about individual medicines Health Tech Assess (Winchester, England) 2007 11 1 160 10.3310/hta11050 17280623

[B19] Kawase A Sembiring ET Choi J Koyama S Hibino H The evaluation of consumers’ attention to risk and benefit information on OTC medicine packages [In Japanese] 2010 Hyogo, Japan: Paper presented at Japan Association for Consumer Studies Conference

[B20] Ministry of Health, Labour and Welfare Statistics on annual population [In Japanese] 2009 http://www.mhlw.go.jp/toukei/saikin/hw/jinkou/suikei09/dl/suikei.pdf

[B21] Ministry of Internal Affairs and Communication Population census of Japan [In Japanese] 2010 http://www.stat.go.jp/data/jinsui/2009np/pdf/gaiyou.pdf

[B22] Kaneko R Ishikawa A Ishii F Sasai T Iwasawa M Mita F Moriizumi R Population projections for Japan: 2006–2055: Outline of results, methods, and assumptions JJP 2008 6 76 11 http://www.ipss.go.jp/webj-ad/WebJournal.files/population/2008_4/05population.pdf

[B23] MacLennan HA Sturdee WD Time for evidence-based labeling of over-the-counter medicines Climacteric 2007 10 179 180 10.1080/13697130701371508 17487644

